# Pro-Inflammatory Cytokines Differentially Induce Intercellular Tunneling Nanotube Conduits and Cellular Migration in Pancreatic, Breast, and Colorectal Cancer Cells

**DOI:** 10.3390/biom16020292

**Published:** 2026-02-12

**Authors:** Leili Baghaie, David A. Bunsick, Elizabeth Skapinker, Emilyn B. Aucoin, Abdulrahman M. Yaish, Yunfan Li, Izzah Wahab, Emma Negrea, Milda Gutauskaite, Tashai Berwick-Gardner, Kate Matys, William W. Harless, Myron R. Szewczuk

**Affiliations:** 1 Department of Biomedical & Molecular Sciences, Queen’s University, Kingston, ON K7L 3N6, Canada; 16lbn1@queensu.ca (L.B.); david.bunsick@umassmed.edu (D.A.B.); 18yl210@queensu.ca (Y.L.); 19iw4@queensu.ca (I.W.); 2 Faculty of Arts and Science, Queen’s University, Kingston, ON K7L 3N9, Canada; 21ess18@queensu.ca (E.S.); tashai.berwickgardner@queensu.ca (T.B.-G.); 21kam49@queensu.ca (K.M.); 3 Faculty of Science, Biology (Biomedical Science), York University, Toronto, ON M3J 1P3, Canada; emilyn08@my.yorku.ca; 4 Faculty of Health Sciences, Queen’s University, Kingston, ON K7L 3N9, Canada; a.yaish@queensu.ca (A.M.Y.); 21enn2@queensu.ca (E.N.); 20mg71@queensu.ca (M.G.); 5 ENCYT Technologies Inc., Membertou, NS B1S 0H1, Canada

**Keywords:** pro-inflammatory cytokines, tunneling nanotube, cellular cancer migration, pancreatic, breast, colorectal cancer cell lines

## Abstract

**Background**: When tumors are surgically removed, an immediate rise in circulating tumor cells is often observed, accompanied by several postoperative changes that can enable these cells to evade immune detection and metastasize. The perioperative period following tumor resection can often promote the formation of new distant micrometastatic foci triggered by upregulation of distinct molecules. Our lab previously reported an increase in distinct inflammatory cytokine molecules following surgical resection in prostate, breast, and colorectal cancer patients, and the secretion of these signals begins as early as 2–24 h after surgery. Here, we investigated whether these distinct cytokines could orchestrate the formation of tunneling nanotube (TNT) conduits to enhance cancer cell migration. **Methods and Results**: Here, we provide supporting evidence that specific pro-inflammatory cytokines upregulated following cancer surgery may be potential triggers of disease recurrence and migration through TNT formation. In the tumor microenvironment, TNTs act as conduits between cancer and normal cells, facilitating the transfer of organelles that contribute to cancer cell survival and metastasis. Here, The effects of TGF-β1, IL-6, and HGF cytokines on the development of TNT conduits between adjacent cancer cells, as well as the effects of oseltamivir phosphate (OP) treatment, were measured using fluorescent microscopy and image analysis software. In PANC-1 pancreatic cancer cells, the addition of these cytokines significantly increased (*p* < 0.009) the quantity and extent of TNTs compared with untreated control cells. MCF-7 breast cancer cells yielded comparable results, with a significant increase in TNT observed in cells treated with TGFβ-1, IL-6, and HGF. In contrast, SW620 colorectal cancer cells did not express TNTs in response to any of the three cytokines tested. OP treatment with cytokines significantly reduced TNT formation in pancreatic and breast cancer cells, with no effect on the colorectal SW620 cancer cell line. Cell migration in response to cytokines was assessed using the scratch wound assay. Out of the three cell lines analyzed, the PANC-1 cells fully closed after 12 h of the wound gap. In contrast, the SW620 and MCF-7 cells had no significant change in wound closure rate following cytokine treatment. The SW620 cells exhibited a slight but insignificant increase in the wound closure rate with TGFβ-1 and HGF treatment, while IL-6 in the SW620 cells and all three cytokines in the MCF-7 cells were comparable to the control. OP significantly reduced the scratch wound closure rate on PANC-1, SW620, and MCF-7 cells treated with these cytokines. **Conclusions**: These findings further support the link between perioperative cytokine activity and increased metastatic potential by promoting the formation of intercellular tunneling nanotube conduits. OP, a specific inhibitor of the mammalian neuraminidase-1 (NEU-1) enzyme, disrupts this process.

## 1. Introduction

Tunneling nanotubes (TNTs) are thin, membrane-enclosed channels that connect tumor cells and tumor microenvironment cells, enabling the transfer of critical metabolites and molecules such as mitochondria, proteins, and RNA [1]. This networking facilitates cancer cells’ acquisition of new metastatic traits, such as increased resistance to chemotherapy, enhanced metabolic activity, and enhanced migration, all of which promote tumor aggressiveness, metastasis, and disease progression. Recently, Chen and Zhao [2] provided a comprehensive review on the molecular composition, structural characteristics, and functional roles of TNTs in cancer. The essential approach is to understand the structural and functional heterogeneity of TNTs across different tumor cells and how this cell heterogeneity affects TNT function. The physiological status of the cancer cell also determines the nature and magnitude of the TNT communication, i.e., whether the interacting cells are under normal or stressed conditions [3].

We recently reported on a distinct immunologic and angiogenic cytokine profile following surgical resection of breast, colorectal, and prostate cancers [4]. Immediate increases in MMP-9, IL-6, and HGF paired with decreases in angiogenic growth factors such as VEGF, FGF2, TGF-β1, PDGF-AB/BB, and growth factors like IGF-1 and EGF were noted. While most of these cytokines return to baseline levels rapidly (48–96 h), this brief inflammatory state may be sufficient to facilitate metastasis in a surviving cancer cell population after surgery through distinct biological processes.

Baghaie et al. [5] reported that TGF-β1, HGF, and IL-6 increase the expression of mesenchymal N-cadherin and vimentin markers in PANC-1, SW620, and MCF-7 cell lines while reducing epithelial E-cadherin markers, consistent with the EMT process. Interestingly, the report found that these cytokine receptors downstream of EMT signaling were regulated by neuromedin B receptor (NMBR)–matrix metalloproteinase 9 (MMP9)–neuraminidase 1 (NEU-1) crosstalk tethered to the receptors, suggesting that these cytokine receptors drive increased NEU-1 activity and subsequent signaling pathway activation. Oseltamivir phosphate (OP) was found to inhibit NEU-1 activity induced by cytokine-binding receptors, leading to downregulation of these signaling activations [5].

Here, we investigated whether TGF-β1, IL-6, and HGF, binding to their respective cytokine receptors in combination with OP treatments, affect intercellular TNT communication conduits to enhance cellular migration in pancreatic PANC-1, colorectal SW620, and breast MCF-7 cancer cell lines. The selection of these cancer cell lines was based on the observed dramatic changes with TGFβ-1, IL-6, and HGF levels in colorectal and breast cancer patients following surgical resection [4]. TNTs and cytokines are not mutually exclusive; they are both active forms of communication networks. Cytokines provide a “call to action,” while TNTs enable direct transfer of cellular information through complex intracellular interactions [6]. Pancreatic PANC-1 cancer cells were selected as an aggressive, highly metastatic cancer cell line.

## 2. Materials and Methods

### 2.1. Cell Lines

PANC-1 (human, epithelial morphology, mutation in the KRAS oncogene, TP53 tumor suppressor gene and deletion of the CDKN2A/p16INK4A gene, ATCC CRL-1469TM, Manassas, VA 20110-2209, USA) isolated from pancreatic duct epithelioid carcinoma, SW620 (human, epithelial morphology, rounded morphology with fewer protrusions and a less extended lamellipodial area, ATCC CCL-227TM, Manassas, VA 20110-2209, USA) isolated from the large intestine and colon of colorectal adenocarcinoma, and MCF-7 (human, epithelial morphology, estrogen and progesterone receptor positive, ATCC HTB-22TM, Manassas, VA 20110-2209, USA) isolated from mammary adenocarcinoma were acquired from the American Type Culture Collection (ATCC; Manassas, VA, USA). Cells were grown in culture media containing Dulbecco’s Modified Eagle’s Medium (DMEM) (Gibco, Rockville, MD, USA) supplemented with 10% fetal bovine serum (FBS) (HyClone, Logan, UT, USA) and 5 μg/mL plasmocin (InvivoGen, San Diego, CA, USA) as a prophylactic inhibitor of mycoplasma. Cells with single passage were maintained in an incubator with 5% CO_2_ at 37 °C until 75% confluence was reached.

### 2.2. Cytokines

The three cytokines selected for this study were chosen based on their significant response profiles observed in the patients’ plasma/serum with breast, colorectal, and prostate cancer after surgical resection, as reported by Baghaie et al. [4]. The concentrations of these cytokines resembled the peak levels observed in the same study: the concentrations of the cytokines were Transforming Growth Factor β-1 (TGFβ-1) (Abcam Inc., Cambridge, MA, USA) at 4.0 × 10^−3^ μg/mL, Interleukin-6 (IL-6) (Sigma-Aldrich, St. Louis, MO, USA) at 4.1 × 10^−5^ μg/mL, and Hepatocyte Growth Factor (HGF) (Sigma-Aldrich, St Louis, MO, USA) at 5.97 × 10^−4^ μg/mL. The cytokines were diluted in media without FBS to prevent any confounding interactions between cells and the rich source of proteins and growth factors present in FBS.

### 2.3. Inhibitors

Oseltamivir phosphate (OP) (>99% pure OP, batch No. MBAS20014A, Solara Active Pharma Sciences Ltd., New Mangalore-575011, Karnataka, India), a broad-spectrum sialidase inhibitor that inhibits NEU-1, was used at a concentration of 300 μg/mL as previously reported by Baghaie et al. [5].

### 2.4. Tunneling Nanotubes (TNTs)

PANC-1, SW620, and MCF-7 cells were grown in a culture medium containing 10% FBS at a density of 200,000 cells/well on 12 mm circular glass coverslips in a 24-well plate and incubated at 37 °C until 75% confluence was reached. Adherent cells were then treated with cytokines in DMEM without FBS (see Section 2.2 Cytokines) for 24 h, while control cells were incubated with FBS-free media for the same duration. For OP testing, 150 μL of the 300 μg/mL compound was added 15 min prior to cytokine addition. Cells were then washed and subsequently treated with CellMask^TM^ Orange Actin Tracking Stain with excitation at 545 nm (A57244, Invitrogen, Thermo Fisher Scientific Inc., Waltham, MA, USA) and fixed with 4% PFA before being incubated at 4 °C for 24 h. The following day, cells were washed, and coverslips with attached cells were inverted onto a slide with Vectashield DAPI fluorescent mounting medium (VECTH1500, MJS BioLynx Inc., Brockville, ON, Canada) to stain the nuclei. Slides were immediately visualized using Zeiss M2 epi-fluorescent microscopy (20 × objective magnification, Carl Zeiss Canada Ltd., Toronto, ON, Canada). Cell projections were differentiated from the rest of the cell body using CellProfiler and ImageJ (Version 23.1.0.389). Fluorescence density was quantified further with Corel Photo-Paint X8, Version 24.3.0.57, using the same density equation as previously reported by Baghaie et al. [5].

### 2.5. Scratch Wound Assay

Cells at 50,000 cells/well were plated in Ibidi cell culture 2-well silicone insert with a defined 500 µm cell-free gap on an ibiTreat #1.5 polymer coverslip, tissue culture treated, sterilized μ-Dish 35 mm and were incubated for 24 h at 37 °C in a 5% CO_2_ incubator. PANC-1, SW620, and MCF-7 cells were seeded in the thin-bottom coverslip dish in culture media and allowed to adhere to 90% confluence in a 37 °C, 5% CO_2_ incubator. A sterile 10 μL pipette tip was used to create a wound, and non-adherent cells were removed using a medium wash. The control cells were then supplemented with media containing 5% FBS, while the cytokine treatment cells were supplemented with either TGFβ-1, IL-6, or HGF in media without FBS. The OP-treated cells were incubated with 300 µg/mL OP for 15 min before cytokine addition. Imaging was performed using a Nikon Eclipse Ti2 microscope (4× objective magnification, Nikon Instruments Inc., Melville, NY, USA) at hourly intervals for the first 6 h, then at 12 h after the wound was created. The wound width was measured at 6–8 points per image using the NIS-Elements AR software, Version 5.21.00, and used to fit a simple linear regression model in GraphPad Prism 10, which yielded the rate of wound closure in μm/h.

### 2.6. Statistical Analysis

All statistical analyses were performed using GraphPad Prism 10 software and are presented as the mean ± standard error of the mean (*SEM*). Comparisons between groups from two to three independent experiments were made by one-way analysis of variance (ANOVA) at 95% confidence using the uncorrected Fisher’s LSD multiple comparisons test with 95% confidence. Asterisks denote statistical significance.

## 3. Results

### 3.1. TGFβ-1, IL-6 and HGF Induce Intercellular Tunneling Nanotube Conduits in PANC-1, SW620 and MCF-7 Cancer Cells

**Figure 1:**
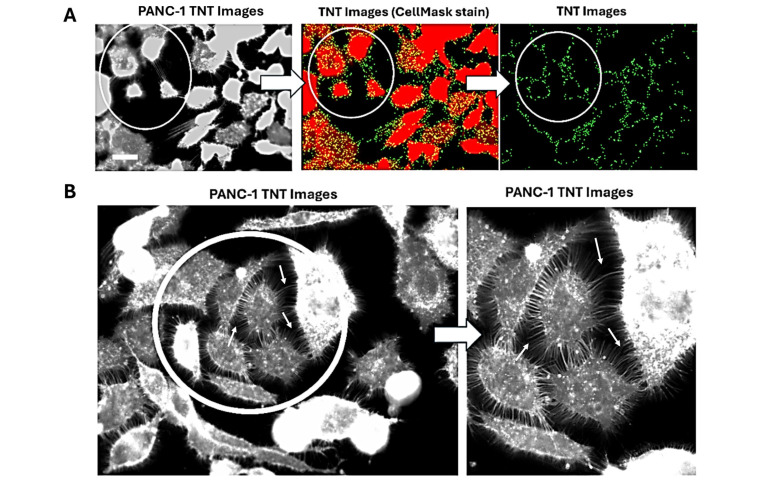

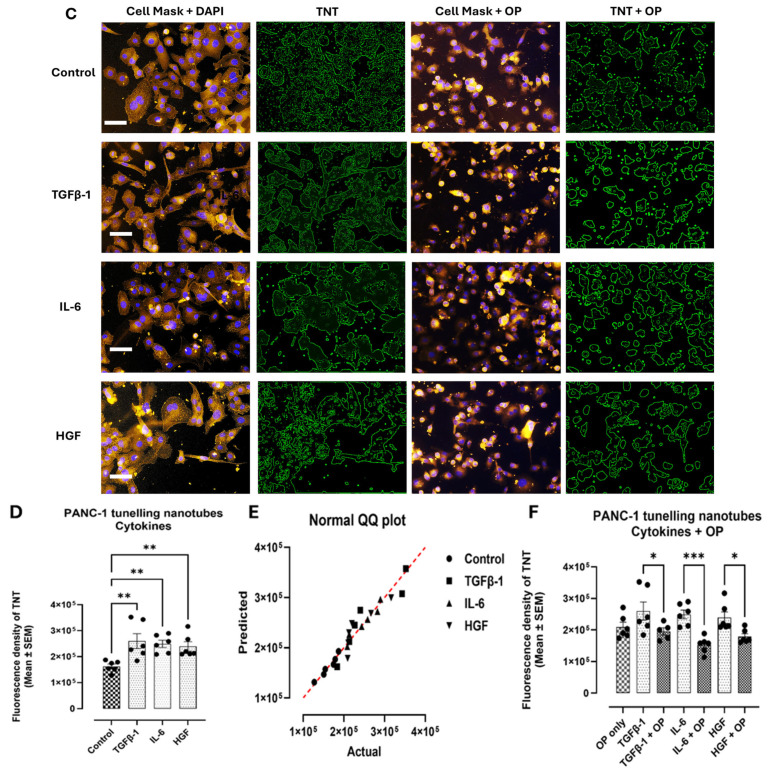
Cytokine treatment increases the number of tunneling nanotubes, while cytokines with OP decrease their quantity in PANC-1 cells. (**A**) Images of PANC-1 cells depicting an example of TNT analysis. (**B**) The image reveals an enlarged extent of microtube conduits between cancer cells (arrows). (**C**) PANC-1 cells were treated with either TGFβ-1 (4.0 × 10^−3^ μg/mL), IL-6 (4.1 × 10^−5^ μg/mL), or HGF (5.97 × 10^−4^ μg/mL), or media without FBS (control) for 24 h prior to being stained with a plasma membrane cell mask (excitation at 554 nm). Cell mask and DAPI nuclear staining are shown alongside TNT images (green). Imaging of stained cells demonstrated that the addition of all three cytokines increased the number of cellular extensions (tunneling nanotubes) (**C**,**D**). The addition of OP significantly decreased cytokine-induced TNTs (**C**,**F**). Images were captured using Zeiss Imager M2 epifluorescent microscopy (cell + OP, scale bar: 40× magnification; cell, scale bar: 40× magnification). Quantification of these extensions using fluorescence density (**D**), represented as mean ± SEM (*n* = 6, independent experiments 2–3), F (3, 20) = 5.583, *p* = 0.0060, significantly increased the amount and xplainedlength of extensions (*p* < 0.009). (**E**) A normal QQ plot assesses the normality of a dataset, assuming it follows a normal distribution. In contrast, the addition of OP (**C**,**F**) decreased tunneling nanotubes when combined with TGFβ-1, IL-6, and HGF (*p* = 0.0121, *p* = 0.0002, *p* = 0.0142, respectively), F (6, 34) = 5.716, *p* = 0.0003. As indicated by asterisks, statistical significance was calculated with ANOVA and Fisher’s uncorrected LSD post hoc test at a confidence level of 95%, *** *p* < 0.001, ** *p* < 0.01 and * *p* < 0.05. *Abbreviations*: TGFβ-1: Transforming growth factor beta-1; IL-6: interleukin-6; HGF: hepatocyte growth factor; OP: Oseltamivir phosphate.

To determine the extent of cytokine-induced cellular interaction networks, a tunneling nanotube (TNT) assay was performed. These nanotubes extend from the plasma membrane of cells, allowing for cell-to-cell contact over long distances. They have also been implicated in cancer, where they are given the name “tumor microtubes” and aid in cancer progression. Figure 1A shows TNT projections in green after analyses using Image J. Figure 1B depicts example enlarged images showing tumor microtubes (arrows) on PANC-1 cells in culture. To investigate cytokine effects on cancer cell lines, pancreatic, colorectal, and breast cancer cells were treated with either TGFβ-1 (4.0 × 10^−3^ μg/mL), IL-6 (4.1 × 10^−5^ μg/mL) or HGF (5.97 × 10^−4^ μg/mL), or media without FBS (control) for 24 h before being stained with a plasma membrane cell mask (excitation at 554 nm) (Figure 1C). The three cytokines selected for this study were chosen based on their significant alterations observed in the plasma profiles of breast, colorectal, and prostate cancer patients after surgical resection, as reported by Baghaie et al. [4]. The concentrations of these cytokines were also shown to induce EMT in these same cells, as reported by Baghaie et al. [5].

With PANC-1 cells, the addition of cytokines significantly increased (*p* < 0.009) the quantity and extent of TNTs compared with untreated control cells (Figure 1D). Figure 1E shows the normality test to determine if a dataset follows a normal distribution. To assess normality visually, a QQ plot of the data against expected values from a normal distribution indicates that the dataset is approximately normally distributed.

In addition, Baghaie et al. [5] reported that TGFβ-1, IL-6, and HGF cytokines transactivate their receptors on cancer cells to induce EMT, and that this process is regulated by neuraminidase-1 (NEU-1). They found that oseltamivir phosphate (OP) prevented NEU-1-mediated sialic acid hydrolysis of cytokine receptors, leading to significant downregulation of these receptors’ signaling cascades and reduced EMT phenotypes [5]. Based on these reported findings, here, we hypothesized that OP downregulates the TNT formation induced by TGFβ-1, IL-6, and HGF. Figure 1C,F show a significant downregulation of TGFβ-1, IL-6, and HGF induced TNT formation pretreated with OP for 15 min. It is noteworthy that OP inhibition did not cause cytotoxicity in cells, as reported by Baghaie et al. [5].

**Figure 2:**
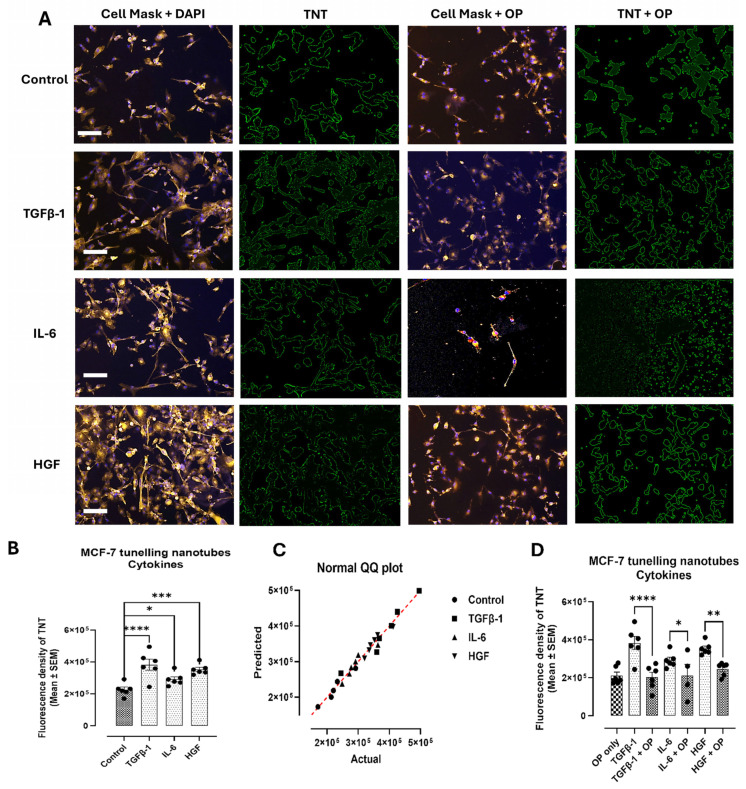
Cytokine treatment increases the number of tunneling nanotubes, while cytokines with OP decrease their quantity in MCF-7 cells. (**A**) MCF-7 breast cancer cells were treated with either TGFβ-1 (4.0 × 10^−3^ μg/mL), IL-6 (4.1 × 10^−5^ μg/mL) or HGF (5.97 × 10^−4^ μg/mL), or media without FBS (control) for 24 h prior to being stained with a plasma membrane cell mask (excitation at 554 nm). Nuclear staining is shown with DAPI. Imaging of stained cells (**A**) demonstrated that the addition of all three cytokines increased the number of cellular extensions (tunneling nanotubes), whereas the addition of OP decreased cytokine-induced TNTs. Images were captured using a Zeiss Imager M2 epifluorescent microscope (40× magnification, scale bar). (**B**) Quantification of these extensions using fluorescence density represented as mean ± SEM (n = 6, independent experiments 2) significantly increased the amount and length of extensions (TGFβ-1 *p* < 0.0001; IL-6 *p* = 0.0448; HGF *p* = 0.0005), F (3, 20) = 10.37, *p* = 0.0002. (**C**) A normal QQ plot assesses the normality of a dataset and is expected to follow a normal distribution. (**D**) The addition of OP decreased TNTs when combined with TGFβ-1, IL-6, and HGF (*p* < 0.0001, *p* = 0.0418, *p* = 0.0035, respectively), F (7, 38) = 7.980, *p* < 0.0001. As indicated by asterisks, statistical significance was calculated with ANOVA and Fisher’s uncorrected LSD post hoc test at a confidence level of 95%. **** *p* < 0.0001, *** *p* < 0.001, ** *p* < 0.01, and * *p* < 0.05.

MCF-7 cells yielded comparable results to the PANC1 cells, with a significant increase in TNT fluorescence density observed in TGFβ-1, IL-6, and HGF-treated cells (*p* < 0.0001, *p* = 0.0448, and *p* = 0.0005, respectively) (Figure 2A,B). Pretreated MCF-7 cells with OP for 15 min, and the cytokines revealed a significant downregulation of the TNTs.

**Figure 3:**
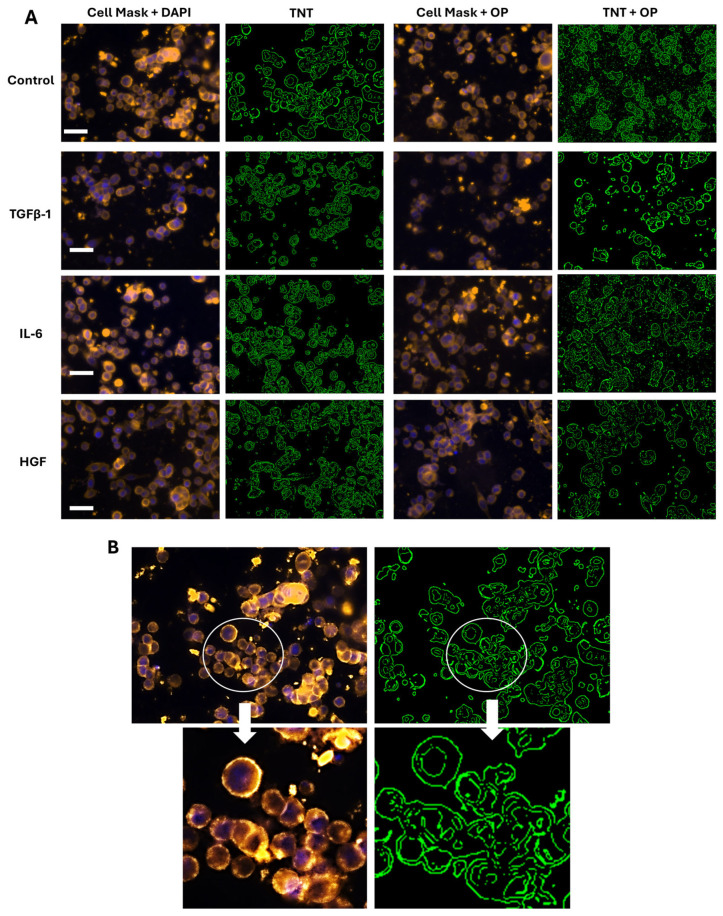
SW620 cells do not express tunneling nanotubes at the highest magnification. (**A**) Colorectal cancer cells (SW620) were treated with either TGFβ-1 (4.0 × 10^−3^ μg/mL), IL-6 (4.1 × 10^−5^ μg/mL) or HGF (5.97 × 10^−4^ μg/mL), or media without FBS (control) for 24 h or OP (300 μg/mL) for 15 min prior to the addition of the same concentration of cytokines as part A. Cells were stained with cell mask plasma membrane (excitation at 545 nm). Nuclear staining with DAPI was also done. Images were captured using a Zeiss Imager M2 epifluorescent microscope (40× magnification, scale bar). Imaging of stained sections demonstrates the absence of tunneling nanotubes, including the untreated control at the highest microscope magnification (40× objective), representing one out of two independent experiments. (**B**) The control image was enlarged to reveal the extent of TNTs.

In contrast, SW620 cells did not express TNTs in response to any of the cytokines tested (Figure 3). Indeed, SW-620 cells have been reported to have a rounded morphology with fewer protrusions and a less extended lamellipodial area, a membrane protrusion at the leading edge of cells that drives cell migration in many normal and pathological situations [7].

### 3.2. TGFβ-1, IL-6 and HGF Enhance the Migratory Potential of Pancreatic PANC-1 Cancer Cells Only in a Scratch Wound Assay

**Figure 4:**
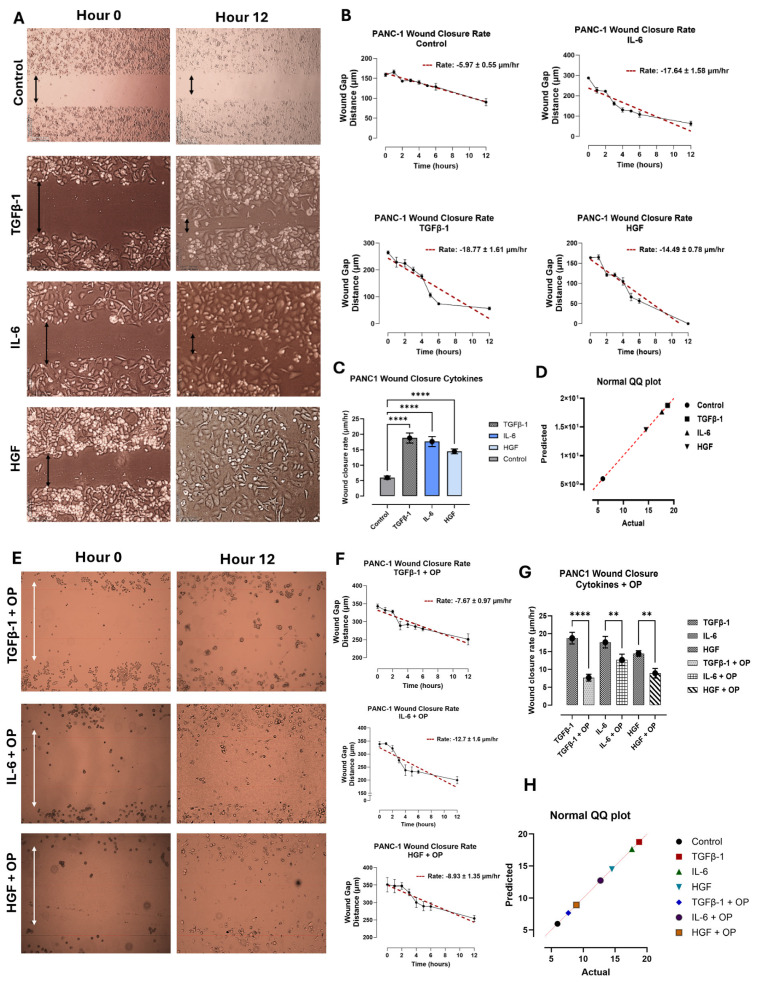
Cytokine treatment increases wound closure rate in PANC-1 cells. Pancreatic cancer cells (PANC-1) were treated with either TGFβ-1 (4.0 × 10^−3^ μg/mL), IL-6 (4.1 × 10^−5^ μg/mL), or HGF (5.97 × 10^−4^ μg/mL), or media with FBS (control). A scratch wound was created using a sterile pipette tip, and the wound closure rate was measured hourly for 6 h, then subsequently at 12 h following the creation of the wound. (**A**) Imaging was taken with a Nikon Eclipse Ti2 microscope (4× magnification) every hour for the first 6 h, and 12 h after the creation of the scratch wound. The wound width was measured at 6–8 points per image using the microscope NIS-Elements AR software, version 5.21.00, and the results were analyzed to create a simple linear regression (red dashed line) (**B**). To determine the significance of the wound closure rate of the cytokine-treated cells compared to untreated controls, a one-way analysis of variance (ANOVA) was used (**C**), with each bar representing the mean ± SEM (obtained from B) (*p* < 0.0001), F (3, 182) = 22.66, *p* < 0.0001. (**D**) A normal QQ plot assesses the normality of a dataset and is expected to follow a normal distribution. (**E**) The addition of 300 μg/mL OP to cytokine-treated cells significantly reduces the rate of wound closure. (**F**) The addition of OP 15 min prior to cytokine treatment significantly decreases wound closure rate and alters the morphology of colorectal cancer cells (**G**). Comparison of cytokine-treated cells with cells treated with cytokine and OP is a representation of one of three separate experiments with similar results, using the mean ± SEM (obtained from F); F (6, 317) = 15.55, *p* < 0.0001. As indicated by asterisks, statistical significance was calculated with ANOVA and Fisher’s uncorrected LSD post hoc test at a confidence level of 95%, **** *p* < 0.0001, ** *p* < 0.01. One-way analysis of variance (ANOVA) shows a significant decrease in wound-healing rate across all treatment groups (*p* < 0.0001). (**H**) A normal QQ plot assesses the normality of a dataset and is expected to follow a normal distribution.

PANC-1, SW620, and MCF-7 cells were treated with either TGFβ-1 (4.0 × 10^−3^ μg/mL), IL-6 (4.1 × 10^−5^ μg/mL), or HGF (5.97 × 10^−4^ μg/mL), or media with 5% FBS (control). A wound was created on the cell surface using a sterile pipette tip, and the gap width was immediately measured to give the baseline (hour 0). The wound width was continuously measured hourly for the first 6 h, and then at 12 h after wound creation. The data were analyzed to estimate wound closure rate (μm/h) using simple linear regression. The findings demonstrate that only the pancreatic PANC-1 cancer cells had a significant increase in the wound closure rate with the cytokine-treated cells compared to the untreated control (*p* < 0.0001) (Figure 4). Out of the three cell lines analyzed, the PANC-1 cells fully closed after 12 h of the wound gap.

**Figure 5:**
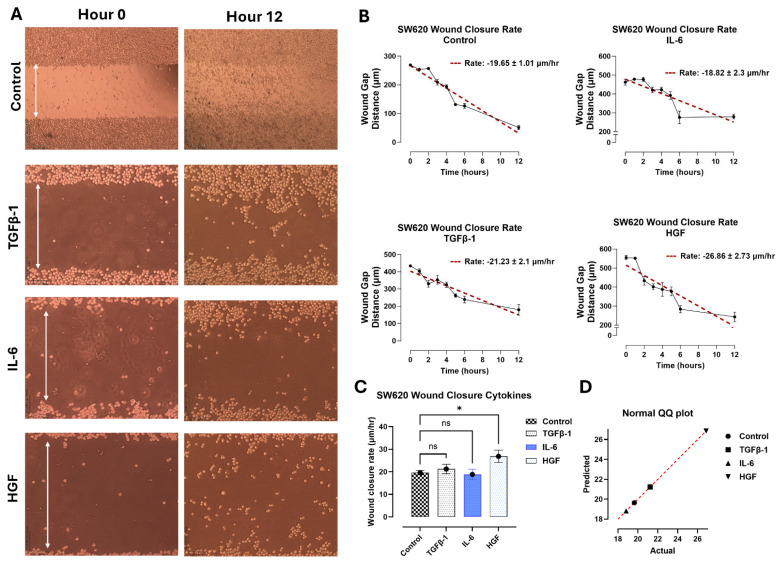

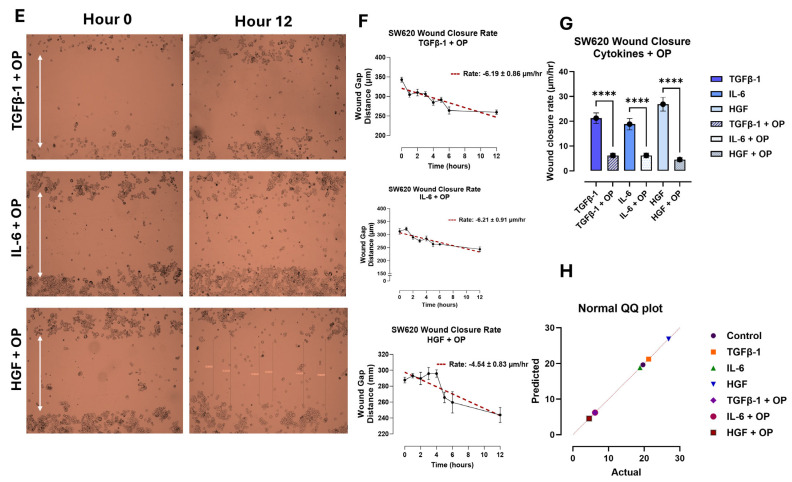
SW620 cells were treated with either TGFβ-1 (4.0 × 10^−3^ μg/mL), IL-6 (4.1 × 10^−5^ μg/mL), or HGF (5.97 × 10^−4^ μg/mL), or media with FBS (control). A scratch wound was created using a sterile pipette tip, and the wound closure rate was measured hourly for 6 h, then subsequently at 12 h following the creation of the wound. (**A**) Imaging of the wound using a Nikon Eclipse Ti2 microscope (4× objective magnification) shows the wound width at hours 0 and 12. The rate of wound closure (μm/h) was quantified using simple linear regression for untreated cells, TGFβ-1-Treated Cells, IL-6-treated cells, and HGF-treated cells (**B**). (**C**) Comparison of cytokine-treated cells with untreated control cells is shown as the mean ± SEM (obtained from B), and one-way analysis of variance (ANOVA) shows no significant difference, except for HFG, F (3, 182) = 2.867, *p* = 0.0380. (**D**) A normal QQ plot assesses the normality of a dataset, assuming it follows a normal distribution. (**E**) The addition of 300 μg/mL OP to cytokine-treated cells significantly reduces the rate of wound closure. (**F**) The addition of OP 15 min prior to cytokine treatment significantly decreases wound closure rate and alters the morphology of colorectal cancer cells (**G**). Comparison of the rates of cytokine-treated cells compared to cells treated with cytokine and OP is a representation of one out of three separate experiments with similar results using the mean ± SEM (obtained from F), and one-way analysis of variance (ANOVA) demonstrates a significant decrease in wound healing rate in all treatment groups (*p* < 0.0001), F (6, 314) = 26.39, *p* < 0.0001. (**H**) A normal QQ plot assesses the normality of a dataset, assuming it follows a normal distribution. As indicated by asterisks, statistical significance was calculated with ANOVA and Fisher’s uncorrected LSD post hoc test at a confidence level of 95%. ns = non-significant, **** *p* < 0.0001, and * *p* < 0.05.

**Figure 6:**
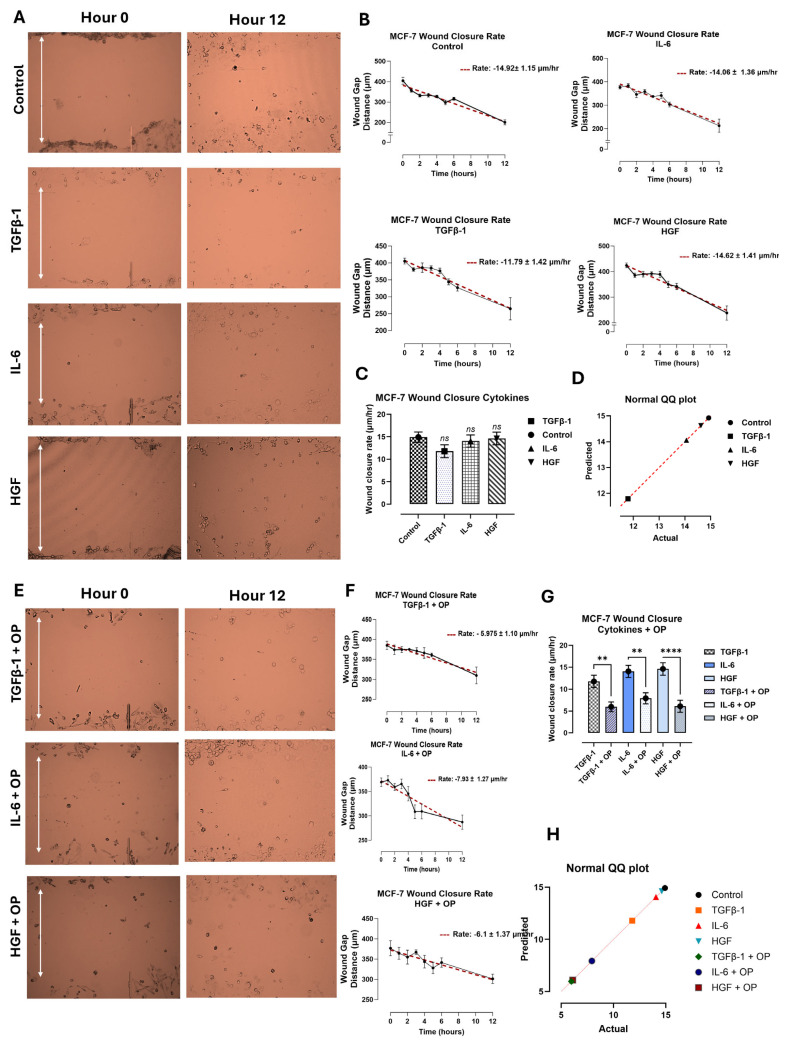
MCF-7 cells were treated with either TGFβ-1 (4.0 × 10^−3^ μg/mL), IL-6 (4.1 × 10^−5^ μg/mL), or HGF (5.97 × 10^−4^ μg/mL), or media with FBS (control). A scratch wound was created using a sterile pipette tip, and the wound closure rate was measured hourly for 6 h, then subsequently at 12 h following the creation of the wound. (**A**) Imaging of the wound using a Nikon Eclipse Ti2 microscope (4× objective magnification) shows the wound width at hours 0 and 12. The rate of wound closure (μm/h) was quantified using simple linear regression for untreated cells, TGFβ-1-Treated Cells, IL-6-treated cells, and HGF-treated cells (**B**). (**C**) Comparison of cytokine-treated cells with untreated control cells is shown as the mean ± SEM (obtained from B), and one-way analysis of variance (ANOVA) shows no significant difference, F (3, 181) = 1.115, *p* = 0.3445. (**D**) A normal QQ plot assesses the normality of a dataset and is expected to follow a normal distribution. (**E**) The addition of 300 μg/mL of OP to cyto-kine-treated cells significantly decreases the wound closure rate. (**F**) The addition of OP 15 min prior to cytokine treatment significantly decreases wound closure rate and alters the morphology of colorectal cancer cells (**G**). Comparison of the rates of cytokine-treated cells compared to cells treated with cytokine and OP is a representation of one out of three separate experiments with similar results using the mean ± SEM (obtained from F), and one-way analysis of variance (ANOVA) demonstrates a significant decrease in wound healing rate in all treatment groups (*p* < 0.0001), F (6, 310) = 9.215, *p* < 0.0001. (**H**) A normal QQ plot assesses the normality of a dataset and is expected to follow a normal distribution. As indicated by asterisks, statistical significance was calculated with ANOVA and Fisher’s uncorrected LSD post hoc test at a confidence level of 95%. ns = non-significant, **** *p* < 0.0001, and ** *p* < 0.01.

In contrast, the SW620 cells (Figure 5) and MCF-7 cells (Figure 6) had no significant change in wound closure rate following cytokine treatment. The SW620 cells exhibited a slight but nonsignificant increase in wound closure rate with TGFβ-1 and HGF treatment (Figure 5). In contrast, IL-6 in SW620 cells was significantly increased, whereas the other three cytokines in MCF-7 cells were comparable to the control (Figure 6). It is noteworthy that SW620 and MCF-7 cells were observed slightly detaching from the outer surface of the wound and entering the wound gap space approximately after 4 h; however, they did not appear to impact wound closure.

Another aspect of interest in OP treatment and inhibition results was its role in cellular migration in a scratch wound assay. Here, cells were first treated with 300 μg/mL of OP for 15 min before the addition of the cytokines. The wound closure rate (μm/h) was significantly decreased in PANC-1 cells treated with TGFβ-1 + OP, HGF + OP, and IL-6 + OP (Figure 4E–G) compared to those treated with cytokines only. SW620 (Figure 5) and MCF-7 cells (Figure 6) demonstrated a significant reduction in wound closure rate in all three cytokines plus OP treated groups (MCF-7: (TGFβ-1; *p* = 0.0023), (IL-6; *p* = 0.0011), (HGF; *p* < 0.0001); SW620: *p* < 0.0001). These findings directly support our previous findings of the inhibitory and decreased proliferative capabilities of OP, as reported by Baghaie et al. [5].

## 4. Discussion

The cellular and metabolic activities of cancer cells are highly regulated by tunneling nanotube (TNT) conduits. These TNTs are intercellular network conduits used by both cancer and normal immune cells to facilitate the transfer of calcium waves between cells, mitochondria, lysosomes, and proteins [3]. The metabolic activity of cancer cells is integrated and mutually regulated by these tunneling nanotube communications. Various cytokines and chemokines are produced by tumor cells, cancer-associated fibroblasts, and tumor-associated immune cells in the tumor microenvironment (TME), which stimulate EMT and thereby promote cancer metastasis [5,8]. EMT in cancer development is characterized by the loss of intercellular adhesion structures, alterations in cell polarity, and a transition to a spindle-shaped cell morphology [9]. This process involves the downregulation of intercellular connections, a switch from keratin to vimentin intermediate filaments, and an enhancement of cell invasion and motility [9].

TNTs, in turn, have been reported to contribute to survival, metastasis, and chemoresistance in cancer cells [3]. Here, the in vitro effects of TGFβ-1, IL-6, and HGF on human pancreatic PANC-1 and breast MCF-7 cancer cell lines significantly altered cancer cells, forming missile-like shapes that induced tunneling nanotube (TNT) conduits. In contrast, colorectal SW-620 cancer cells did not form TNTs following this cytokine treatment and lacked missile-like shapes. The MCF-7 cells retain several characteristics of differentiated mammary epithelium. Both PANC-1 and SW-620 are metastatic, poorly differentiated cell lines. SW-620 cells were derived from a metastatic lymph node of a patient with Dukes C colorectal cancer [10].

Moreover, these cytokines appear to impact cellular migration during wound healing in pancreatic cancer cells (Figure 4, Figure 5 and Figure 6). We previously reported that there is an immediate increase in serum cytokine secretion following surgical resection in breast, colorectal, and prostate cancer patients as part of the wound-healing response [4]. Given that surgical resection remains one of the most common techniques for cancer therapy, the wound assay results in PANC-1, in conjunction with our previous report, suggest that these inflammatory cytokines may contribute to the increased proliferation and invasiveness of residual cancer cells.

In contrast, the cytokine-treated PANC-1 results did not align with the colorectal or breast cancer cells tested. Importantly, in both cases, the SW620 and MCF-7 cells were seen detaching from the outer surface of the wound and entering the wound gap space after approximately 4 h; however, the wound did not close. While the initial conclusions suggest that inflammatory cytokines have no impact on the wound-healing response in these cells, the cells’ nature may be responsible. SW620 cells are derived from a lymph node metastasis of colorectal adenocarcinoma, suggesting that their metastatic potential may be slightly lower than that of PANC-1 cells, which are derived from a primary tumor. Similarly, the MCF-7 cell line is considered less aggressive and invasive, with low metastatic potential [11]. Although MCF-7 cells express estrogen receptors, suggesting that estrogen promotes their growth, in the present study, cells were left untreated to prevent confounding interactions with cytokines, and thus may have had a reduced wound-healing response [12].

Another factor that may have affected the wound-closure rate in colorectal and breast cancer cells is that many of these cytokines act synergistically in vivo. TGFβ-1 has been reported to enhance the inflammatory effects of IL-6 through crosstalk between the SMAD and STAT3 pathways [13]. Similarly, IL-6 has been linked to preparing hepatocytes to respond more effectively to HGF by increasing c-MET expression, the receptor for HGF. In some cases, IL-6 by itself can induce the expression of HGF [14,15]. As the wound-healing process in vivo is much more complex than the in vitro study reported here, it is plausible that these cells require a combination of cytokines to achieve outcomes comparable to those observed in the PANC-1 wound-healing response.

Nonetheless, this cytokine synergic combination is not consistently observed. Several reports suggested that TGFβ-1 and IL-6 may counteract each other’s effects by mutually inhibiting each other’s induction [16]. Similarly, HGF has been shown to inhibit TGF-β signaling during wound healing, exerting an antagonist effect [17,18]. Hence, determining whether an in vitro “cytokine cocktail” could enhance the outcomes of this study is challenging. However, it presents an intriguing avenue for future exploration and evaluation.

In addition to cytokine-induced cell viability, these cytokines increased the number of tunneling nanotubes (TNTs) in PANC-1 and SW620 cancer cells, but not in MCF-7 cells. The nanotubes are believed to serve as intercellular communication conduits that can extend up to ten cell diameters or more from the cell body and connect to non-adjacent cells to transport cargo [1,19]. They are also implicated in cancer cell behavior, where they are given the name “tumor microtubes” and have been linked to cancer progression, chemotherapeutic resistance, and metastasis [19]. Previous work by Resnik et al. [20] found that the amount, length, diameter, and elastic properties of TNTs in normal porcine urothelial cells were very different from those in human invasive cancer urothelial cells. Cancerous cells formed many more TNTs with higher elasticity compared to normal urothelial cells. In the current study, a comparison between cancerous and normal pancreatic, colorectal, and breast tissue was not explored; however, the addition of cytokines TGFβ-1, IL-6, and HGF did increase the number of TNTs of PANC-1 (Figure 1A,B) and SW620 (Figure 2A,B) cells compared to untreated control cells. These interesting findings highlight the role of these cytokines in inducing conformational changes in the cellular cytoskeleton, thereby facilitating communication, which may, in turn, indirectly influence their metastatic potential.

The SW620 cells, on the other hand, did not exhibit any tunneling nanotubes, even at 40× magnification, in either the control or cytokine-treated cells (Figure 3). The presence of TNTs in SW620 has been previously noted; however, those studies use much higher magnification (200×) [21]. Therefore, it can be speculated that this trend may also be observed in colorectal cancer cells, but it was not reported in this study.

Treating cells with oseltamivir phosphate (OP) for 15 min before the addition of cytokines was found to have the lowest impact on cell viability (preliminary optimization data). The decision to treat cells with OP before cytokine addition was based on multiple chemotherapeutic studies demonstrating increased treatment efficacy when administered before surgical resection [22,23,24,25]. Baghaie et al. [5] reported the impact of OP in combination with these cytokines on cell viability. They found that, in all experimental conditions, the combination of OP with cytokines decreased cell viability compared to cytokine treatment alone. However, the cell viability was reduced to levels slightly below those of the untreated control cells, demonstrating that OP does not induce cell death [5]. This report suggests that the inhibitory capability of OP reported in the neuraminidase activity assay is due to the presence of senescent cells. This senescent state was further demonstrated by the decline in tunneling nanotube formation in PANC-1 and MCF-7 cancer cells treated with OP. Cells displayed significantly fewer and less extensive tunneling nanotubes when they were treated with OP for 15 min before the addition of the cytokines, compared to cytokine-only-treated cells. These cells also showed a slight morphological change, becoming rounder and smaller compared to the untreated cells. This morphological change was also observed in the wound-healing assay, in which cells treated with OP exhibited reduced migration and wound-healing capacity. In MCF-7 and SW620 cells, co-treatment with OP and cytokines significantly decreased the wound-healing rate (μm/h). Similarly, in PANC-1 cells, the combination of TGFβ-1, HGF, and IL-6 with OP resulted in a significant reduction in wound-healing rate.

## 5. Conclusions

The important findings in this study demonstrate that the cytokines TGFβ-1, HGF, and IL-6, which are rapidly upregulated after surgical resection, induce intercellular tunneling nanotube conduits and cellular migration in pancreatic and breast cancer cells. The colorectal SW620 cells, on the other hand, did not exhibit any tunneling nanotubes in either the control or cytokine-treated cells. The SW620 cells retained a spherical shape, unlike the missile-like shapes exhibited by pancreatic and breast cells. These cytokines can alter the formation of tunneling nanotube (TNT) conduits, affecting the cancer cell migration interaction network by increasing the number of TNT conduits. These properties may lead to the higher metastatic potential of these cells. In addition, OP induced senescence in these cancer cells, as evidenced by decreased tunneling nanotube formation and wound-healing responsiveness. Serendipitously, the findings reveal for the first time a novel OP therapeutic approach to disrupt the metastatic potential of any surviving cancer cell population before or immediately after cancer surgery.

## 6. Patents

M.R.S. reports patents for the use of NEU-1 sialidase inhibitors in cancer treatment (Canadian patent no. 2858,246; United States patent no. US2015/0064282 A1; European patent no. 1874886.2; Chinese patent no. ZL201180076213.7; German patent no. 602011064575.7; Italian patent no. 502020000014650; UK patent no. 2773340; Swedish patent no. 2773340; Spanish patent no. 773340; Switzerland patent no. 2773340; and French patent no. 2773340). M.R.S. reports a patent for using oseltamivir phosphate and analogs thereof to treat cancer (International PCT patent no. PCT/CA2011/050690). W.W.H. and M.R.S. report a patent on a method to improve the effectiveness of anti-cancer therapies by exposing them to an inflammatory stimulus prior to treatment (Canadian patent no. PCT/CA2017/050765, pending). W.W.H. and M.R.S. report a patent on the compositions and methods for cancer treatment (Canadian patent no. PCT/CA2017/050768). W.W.H. and M.R.S. report a patent titled Cancer Treatment and Metastasis Inhibition Using an Anti-Cancer Stem Cell Agent in Combination with a Neu1 Sialidase Inhibitor or a Cytokine Inhibitor After Primary Cancer Treatment (European Patent Number 17830134.7). W.W.H. and M.R.S. report patents 55983477-9CN and CAN_DMS_150056368.1 on the compositions and methods for the treatment of coronavirus infection and respiratory compromise. W.W.H. and M.R.S. have US FDA clinical trial approval to test OP in patients with pancreatic cancer (clinical trial number #173874).

## Data Availability

All data needed to evaluate the paper’s conclusions are present. The preclinical data sets generated and analyzed during the current study are not publicly available but can be obtained from the corresponding author upon reasonable request. The data will be provided following the review and approval of a research proposal, Statistical Analysis Plan, and execution of a Data Sharing Agreement. The data will be accessible for 12 months for approved requests, subject to possible extensions; contact szewczuk@queensu.ca for more information on the process or to submit a request.

## Abbreviations

EMT	Epithelial–mesenchymal transformation
TNTs	tunneling nanotubes
NEU-1	neuraminidase-1
OP	oseltamivir phosphate
CRC	colorectal cancer
BCT	breast-conserving therapy
TNBC	triple-negative breast cancer
IL-	interleukins
IFNs	interferons
TNFs	tumor necrosis factors
CSFs	colony-stimulating factors
TME	tumor microenvironment
EGF	epidermal growth factor
FGF	fibroblast growth factor
VEGF	vascular endothelial growth factor
HGF	hepatocyte growth factor
FGFRs	fibroblast growth factor receptor
PLGF	platelet-derived growth factor
MMP-9	metalloproteinases
PIGF	placental growth factor
RTK	receptor tyrosine kinase
EBP	elastin binding protein
PPCA	protective protein cathepsin A
PKA	protein kinase A
MAPK	mitogen-activated protein kinase
PKB/Akt	protein kinase B
PANC-1	human, epithelial morphology
ATCC CRL-1469TM	
SW620	human, epithelial morphology
ATCC CCL-227TM	
MCF-7	human, epithelial morphology
ATCC HTB-22TM	
TGFβ-1	Transforming Growth Factor β-1
IL-6	Interleukin-6
PFA	paraformaldehyde
BSA	Bovine Serum Albumin
SEM	mean ± standard error of the mean
ANOVA	one-way analysis of variance
FBS	Fetal Bovine Serum
MMP9i	metalloproteinase inhibitor
MET	mesenchymal–epithelial transition
LPS	lipopolysaccharide
